# Uninephrectomy-Induced Lipolysis and Low-Grade Inflammation Are Mimicked by Unilateral Renal Denervation

**DOI:** 10.3389/fphys.2016.00227

**Published:** 2016-06-14

**Authors:** Denis Arsenijevic, Jean-François Cajot, Benoit Fellay, Abdul G. Dulloo, Bruce N. Van Vliet, Jean-Pierre Montani

**Affiliations:** ^1^Division of Physiology, Department of Medicine, University of FribourgFribourg, Switzerland; ^2^National Center of Competence in Research (Kidney.CH)Zurich, Switzerland; ^3^Chemistry/Hematology Laboratory, Fribourg HospitalFribourg, Switzerland; ^4^BioMedical Sciences Division, Faculty of Medicine, Memorial UniversitySt. John's, NL, Canada

**Keywords:** uninephrectomy, unilateral renal denervation, lipolysis, body composition, cytokines, spleen

## Abstract

Uninephrectomy (UniNX) in rats on a fixed food intake leads to increased lipolysis and a low-grade inflammation with an increased subset of circulating cytokines. Because UniNX ablates renal nerves on the side of the removed kidney, we tested the contribution of unilateral renal denervation in the phenotype of UniNX. We compared Sham-operated controls, left nephrectomy (UniNX) and unilateral left kidney denervation (uDNX) in rats 4 weeks after surgery. uDNX did not affect kidney weight and function. In general, the uDNX phenotype was similar to the UniNX phenotype especially for lipolysis in fat pads and increased low-grade inflammation. uDNX led to decreased fat pad weight and increased hormone sensitive lipase and adipocyte triglyceride lipase mRNA levels in epididymal and inguinal adipose tissue, as well as increased circulating lipolysis markers β-hydroxybutyrate and glycerol. Measured circulating hormones such as leptin, T3 and insulin were similar amongst the three groups. The lipolytic cytokines interferon-gamma and granulocyte macrophage colony stimulating factor were increased in the circulation of both uDNX and UniNX groups. These two cytokines were also elevated in the spleen of both groups, but contrastingly they were decreased in fat pads, liver, and kidneys. Both uDNX and UniNX similarly increased noradrenaline content in fat pads and spleen. Melanocortin 4 receptor mRNA levels were increased in the brains of both uDNX and UniNX compared to Sham and may contribute to increased tissue noradrenaline levels. In addition, the farnesoid x receptor (FXR) may contribute to changes in tissue metabolism and inflammation, as anti-inflammatory FXR was decreased in the spleen but increased in other tissues in uDNX and UniNX compared to Sham. In summary, both uDNX and UniNX in rats promote metabolic and immunological alterations by mechanisms that seem to implicate modification of unilateral renal nerve pathways as well as central and peripheral neural pathways.

## Introduction

Decreased kidney function is often associated with disease conditions including obesity, metabolic syndrome, diabetes and inflammation/infection. This decrease in kidney function is usually attributed to the progression of the disease. However, a primary reduction in kidney function may also in itself influence metabolism and inflammation as shown in rats (Zhao et al., 2011; Arsenijevic et al., 2015) and living kidney donors (Ferreira-Filho et al., 2007; Yilmaz et al., 2015).

During the first 4 weeks after uninephrectomy (UniNX) there is a low-level inflammation, which is associated with enhanced lipolysis in fat pads (Arsenijevic et al., 2015). Strikingly, there is an increase in a subset of circulating cytokines including interferon-gamma (IFNγ) and granulocyte colony stimulating factor (GM-CSF). These two cytokines are increased in the spleen of UniNX animals. Contrastingly, other tissues, such as the kidney, liver and fat pads, showed a decrease in cytokine levels. This could be explained by the anti-inflammatory factors SIRT1, a member of the sirtuin family of class III histone deacetylase (Vachharajani et al., 2016), and farnesoid x receptor (FXR), which were decreased in the spleen but elevated in the other mentioned tissues (Arsenijevic et al., 2015). The finding that FXR is increased after UniNX (Gai et al., 2014) suggests that it may play a protective role in following UniNX (Gai et al., 2016).

A number of questions remain concerning the mechanism by which mild reduction in kidney function affects lipolysis and low-grade inflammation. UniNX leads to removal of renal nerves on the side of surgery. Therefore, we decided to test whether removal of renal nerve connections to the brain, by unilateral renal denervation, can contribute to the lipolytic and cytokine phenotype of UniNX. We hypothesized that the loss of unilateral renal nerves could stimulate central pathways known to alter sympathetic outflow to fat pads and organs, which in turn could lead to lipolysis (Song et al., 2005) and low-grade inflammation (Leong et al., 2010; Pavlov and Tracey, 2015).

## Methods

### Animal preparation and experimental protocol

#### Animals and diets

Male Sprague Dawley rats were purchased from Elevage Janvier (Le Genest-St-Isle, France). Rats arrived at 5 weeks of age with an average weight of 160 g/animal. They were placed in individual cages and given pellet food and water *ad libitum*. After a week acclimation period, rats were randomly distributed to be either Sham operated, subjected to removal of the left kidney (UniNX), or subjected to denervation of the left kidney (uDNX). After surgery, animals were put under a fixed food intake (90% of ad lib fed diet) of normal chow paste to ensure that all groups consumed the same amount of calories, as previously described (Arsenijevic et al., 2015).

#### Surgery

Left uninephrectomy and sham surgery and left renal denervation surgery were performed under general anesthesia and a pre/post operative pain relief procedure that was identical to the one described in our previous paper (Arsenijevic et al., 2015). Left renal denervation (uDNX) was carried out by the method described by Kopp et al. (1984). Basically, the left kidney was accessed in the same manner as for the Sham-operated or UniNX. The renal artery was stripped of visible nerves using fine forceps and then painted with a solution of 10% phenol in absolute ethanol. Prior to placing the kidney back into the retroperitoneal cavity, the kidney was swabbed with saline to remove the phenol solution from the denervation site. The incision site was sutured as for the Sham-operated/UniNX animals.

#### Experimental protocol

Rats were placed in individual cages, in a room with a temperature of 22 ± 1°C, with a 12 h light/dark cycle (light 7.00 a.m.–7.00 p.m.) and had free access to water. Body weight was measured daily prior to feeding (9.00 a.m. to 11.00 a.m.). Four weeks after surgery, rats (12 sham, 12 UniNX, and 12 uDNX) were decapitated for immediate blood collection as previously described (Arsenijevic et al., 2015). Animals were placed on ice for peritoneal macrophage collection using pyrogen free phosphate saline buffer (see below). At this time wet tissue weights were measured. For subcutaneous white adipose tissue (SWAT) all fat under the animal's fur and attached to skin was collected, but only the inguinal white adipose tissue (IWAT) was further analyzed. For mesenteric fat (MWAT), all fat attached to gastrointestinal tract was dissected out. Epididymal fat (EWAT) and retroperitoneal fat (RWAT) were removed from their anatomical sites. Whole tissue for EWAT, liver, kidney, spleen, heart, gastrocnemius muscle, and IWAT were frozen in liquid nitrogen. These tissues were crushed in liquid nitrogen. A small sample < 1 g was retained for analysis purposes. The remainder of the tissue was placed with the carcass for body composition analysis. Concerning the brain, hypothalamus and brain stem areas were cut out. The remaining tissue was placed with the carcass. All experimental protocols were approved by the Ethical Committee of the Veterinary Office of Fribourg, Switzerland.

### Body composition

The skull, thorax and abdominal cavity were incised and the gut was cleaned of undigested food. The carcasses were dried for 2 weeks in an oven at 60°C and then homogenized. Body water content was determined by subtracting dry body weight from the body weight prior drying. Body fat was determined using the Sohxlet method from the dry homogenate (Entenman, 1957). Subtracting the fat mass from the dry homogenate allowed us to calculate the fat free dry mass.

### Blood parameters

Blood was collected on ice in plain and anticoagulant-coated tubes and centrifuged at 4°C. Serum and plasma were then kept at −20°C until analyzed as previously described (Arsenijevic et al., 2015). For a complete list of metabolites, hormones analyzed and the provenance of assay kits, see Table 1.

**Table 1:** **Assay kits for metabolites, hormones and cytokines**.

**Assay kit**	**Kit name/Cat. No**.	**Company**
**METABOLITE ASSAY KITS FOR USE ON SYSTEM ROCHE/COBAS 6000 ANALYZER, MODULE COBAS C501**		
Urea	UREAL kit	1
Triglycerides	TRGL kit	1
Cholesterol	Total cholesterol CHOL2 kit	1
HDL	High density lipoprotein HDLC3 kit	1
**METABOLITE PLATE ASSAY KITS**		
Free fatty acid assay kit	Cat. No. K612-100	2
β-hydroxybutyrate assay kit	Cat. No. K623-100	2
Glycerol assay kit	Cat. No. K630-100	2
**HORMONE ELISA OR EIA**		
Aldosterone	EIA kit Cat. No. 10034377	3
Leptin	EIA kit for Mouse/rat Cat. No. A05176	3
Corticosterone	EIA kit Cat. No. 5006553	3
Ghrelin	EIA kit Cat. No. EK-031-31	4
Glucagon	EIA kit Cat. No. EK-028-02	4
Glucagon-like-peptide 1	EIA kit Cat. No. EK-028-11	4
Angiotensin-1	EIA kit Cat. No. EKE-002-01	4
Angiotensin-2	EIA kit Cat. No. EK-002-12	4
Insulin	ELISA kit Cat. No.90060	5
T3 total	EIA kit Cat. No. 07BC1005	6
Noradrenaline	ELISA kit Cat No. CEA907Ge	7
**CYTOKINES ELISA KITS**		
Erythropoietin	ELISA Cat. No. DY959	8
Interleukin(IL)1α	ELISA Cat. No. DY500	8
IL1β	ELISA Cat. No. RLB00	8
IL1RA	ELISA Cat. No. DY480	8
Granulocyte-Macrophage Colony		
Stimulating Factor (GM-CSF)	ELISA Cat. No. DY518	8
C-reactive protein (CRP)	ELISA Cat. No. DY1744	8
IL4	ELISA Cat. No. BMS628MST	9
IL6	ELISA Cat. No. BMS625	9
IL10	ELISA Cat. No. BMS629	9
Tumor Necrosis factor (TNFα)	ELISA Cat. No. 88-7340	9
Interferon-gamma (IFNγ)	ELISA Cat. No. BMS621	9
Acetylation Stimulating Protein (ASP)	ELISA Cat. No.MBS728340	10
Serum Neopterin	ELISA Cat. No. RE59321	11
Cystatin-C	Immunoassay Cat. No. KK-CYC	12

*^1^Roche, Switzerland; ^2^Biovision, Milpitas, CA, 95035, USA; ^3^Cayman Ann Arbor, Michigan, 48108, USA; ^4^Phoenix Europe, D-76133, Karlsruhe, Germany; ^5^ Crystal Chem, IL, USA; ^6^ MP Biomedicals Europe, Illkirch, 67402, France; ^7^ Wuhan USCN Business Co., Ltd., Wuhan, China; ^8^ R&D, Abingdon OX14 3NB, UK; ^9^ eBioscience–San Diego, CA 92121, USA; ^10^ MyBiosource–San Diego, CA 92195, USA; ^11^ IBL Toronto, ON, M3J 2N5, Canada; ^12^ Buhlmann Basel Switzerland*.

### RT-PCR in epididymal/inguinal white adipose tissue (EWAT/IWAT), liver, spleen, heart, and brain

Total RNA was isolated from powder samples for each tissue, then treated with DNase and finally reverse transcribed (Promega) as previously described (Arsenijevic et al., 1997). Samples were then run on a RT-PCR (iQ cycler Bio-Rad) and each sample was normalized to its cyclophilin value. For the list of primers used and their sources, see Table 2.

**Table 2:** **RT-PCR primers**.

**PRIMERS NAME, SEQUENCE, AND ORIGINAL SOURCE OF SEQUENCES**
Adipose triglyceride lipase (ATGL) sense 5-TGTGGCCTCATTCCTCCTAC-3, antisense 5-AGCCCTGTTTGCACATCTCT-3 (Palou et al., 2009)
Hormone sensitive lipase (HSL) sense 5–TCACGCTACATAAAGGCTGCT–3, antisense 5–AGTTCCCTCTTTACGGGTGG–3 (Palou et al., 2009)
CD36 sense 5–GTCCTGCCTGTGTGA–3, antisense 5–GCTCAAAGATGCTCCATTG–3 (Palou et al., 2009)
Melanocortin 4 receptor (MC4R) sense 5- TAT GGT ACT GGA GCG CGT AA-3, antisense 5- TCA GAC GGA GGA TGC TAT GA-3 (Lopez et al., 2010)
Monocyte chemoattractant protein-1 (MCP-1) sense 5-CCTGTTGTTCACAGTTGCTGCC-3, antisense 5-TCTACAGAAGTGCTTGAGGTGGTTG-3 (McTigue et al., 1998)
Regulated on activation, normal T cell expressed and secreted (RANTES) sense 5-CGTGAAGGAGTATTTTTACACCAGC-3, antisense 5-CTTGAACCCACTTCTTCTCTGGG-3 (McTigue et al., 1998)
Alpha2A adrenergic receptor (A2A-ADR) sense 5-ACGGGCATTGTGA-TGGACTC-3, antisense 5-CAGAACCTCTTCCTGGTGTC-3 (Llado et al., 2002)
Cyclophillin sense 5-TCAGGGCTCTTGAAGTCCC-3, antisense 5-CAGAAAATCACAGCAGCCAAC-3 as reference control (Summermatter et al., 2009)

### Western blot analysis

Tissue protein extracts underwent gel electrophoresis and proteins were then transferred to membranes (Arsenijevic et al., 2015). Membranes were pre-incubated with 1% casein (Vectorlab) for 2 h and then rinsed. Membranes were then incubated 2 h with one of the following primary antibodies—uncoupling protein-1 (UCP1) dilution 1/5000 (cat. no. UCP11, Alpha Diagnostics), SIRT1 dilution 1/200 (sc-19857, Santa Cruz), farnesoid x receptor (FXR) dilution 1/200 (sc-13063, Santa Cruz), and beta-actin dilution 1/1000 (Cat No. 4970–Cell Signaling). Secondary antibody LI-COR anti-rabbit (dilution 1/15000) or anti-goat (dilution 1/15000) were used to detect bands (de Bilbao et al., 2009). The signals were visualized with the use of Odyssey Infrared Imaging System (Li-Cor Biosciences, Bad Homburg, Germany).

### Lipogenic enzyme activity assays

Fatty acid synthase (FAS) activity was measured according to the method described by Penicaud (Penicaud et al., 1991). Briefly, frozen tissue powder from EWAT and IWAT were homogenized on ice and treated as previously described (Arsenijevic et al., 2015).

### Circulating cytokines and markers of immune activation

Rat serum ELISA assays for Acetylation stimulating protein (ASP), interleukin (IL)1α, IL1β, IL1receptor antagonist (IL1RA), IL4, IL6, IL10, granulocyte macrophage colony stimulating factor (GM-CSF), interferon-gamma (IFNγ), C-reactive protein (CRP) and tumor necrosis factor alpha (TNFα). Serum Neopterin, a by-product specific of IFNγ-activated macrophages, was also determined by ELISA. For a complete list of cytokine kits and their provenance, see Table 1.

### Cytokine levels in tissues

Tissue cytokine determination was performed, as previously described (Arsenijevic et al., 2006), on tissues from week 4 post UniNX. Briefly, 100 mg of tissue were homogenized with 600 μl of 1% CHAPS (3-[(3-cholamidopropyl) dimethylammonio]-1-propanesulfonate) in RPMI-1640 medium without phenol red (R7509, Gibco) with a polytron homogenizer (Nakane et al., 1992). The supernatant was collected and frozen at −20°C. Cytokines were assayed using immunoassay kits (Table 1), as described previously (Arsenijevic et al., 2006).

### Macrophage intracellular ROS production

Macrophage were isolated from rat peritoneal cavity with pyrogen free phosphate buffered saline and plated into 96 well plates. The capacity of the macrophages to reduce nitro blue tetrazolium (NBT) was used to access their reactive oxygen species production as previously described (Arsenijevic et al., 2015).

### Noradrenaline tissue content

Noradrenaline content was determined by ELISA (see Table 1 for details). Contents are expressed in both ng/g tissue and ng/total tissue weight to take into consideration the changes in total tissue weight. A portion of the total powdered tissue were homogenized in distilled water and measured the same day as according to kit details.

### Data analysis

All data are presented as means ± SEM. Statistical analysis were performed using ANOVA Tukey-Kramer multiple comparison test (2 tail test) using the Instat3 program (GraphPad). A value of *P* < 0.05 was considered as significant.

## Results

### Kidney weight and renal function

**Figure 1:**
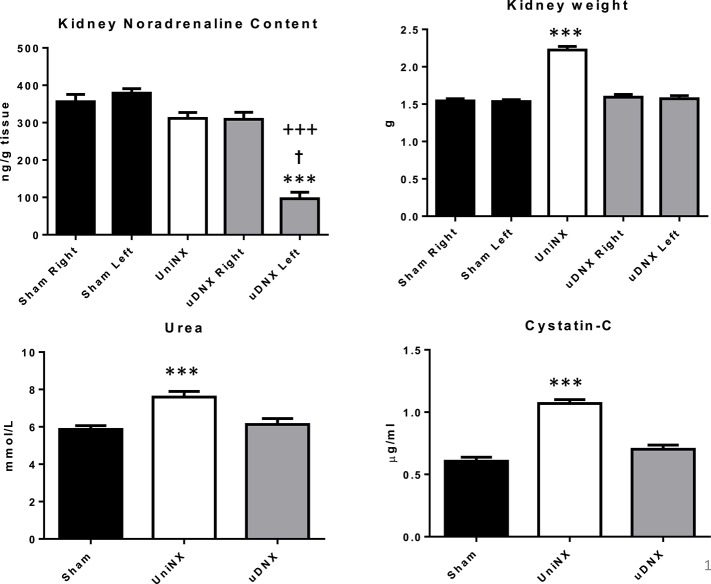
**Week 4 kidney data for Sham, UniNX and uDNX groups; noradrenaline kidney content, kidney weight, and plasma urea and cystatin-C levels**. Values represent means ± SE, *n* = 12/group for plasma and *n* = 8 in tissue. ^***^P < 0.001 corresponds to uDNXL (left) vs. Sham, ^†^*P* < 0.001 uDNXL (left) vs. UniNX, ^+++^*P* < 0.001 uDNXL (left) vs. uDNXR (right) in kidney noradrenaline panel; in other panels ^***^P < 0.001 corresponds to UniNX/uDNX vs. Sham.

UniNX increased the contralateral kidney weight and resulted in a mild reduction in kidney function as evidenced by the increase in both plasma urea and cystatin C levels 4 weeks after surgery compared to Sham operated controls (Figure 1). Unilateral renal denervation (uDNX) did not affect kidney weight or renal function. uDNX resulted in a significant decrease in ipsilateral (left) kidney noradrenaline content 4 weeks after surgery but did not affect the contralateral intact kidney (Figure 1). UniNX did not change noradrenaline levels in the remnant kidney compared to Sham animals.

### Body weight, body composition, and organ weight 4 weeks post-surgery

**Figure 2:**
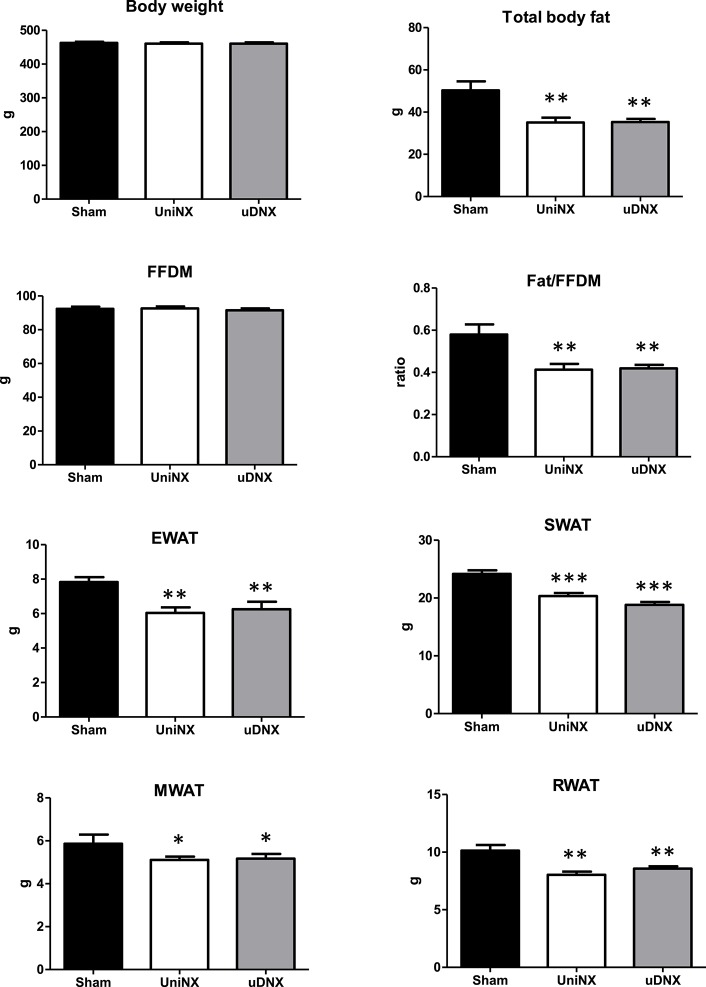
**Week 4 body weight, total body fat, fat free dry mass (FFDM), ratio of body fat/FFDM, epididymal fat (EWAT), subcutaneous fat (SWAT), mesenteric fat (MWAT), and retroperitoneal fat (RWAT) in Sham operated controls, UniNX, and uDNX rats**. Values are means ± SE; *n* = 12/group. ^*^P < 0.05, ^**^P < 0.01, ^***^P < 0.001 corresponds to UniNX/uDNX vs. Sham.

Regarding body composition, both UniNX and uDNX led to a decrease in body fat without affecting fat free dry mass (FFDM; Figure 2). Total body weight was not significantly affected. Decreases in fat pads (epididymal, inguinal, mesenteric and retroperitoneal) were similar in both UniNX and uDNX (Figure 2). UniNX and uDNX liver weights were similar (Sham 17.9 ± 1.3 g, UniNX 18.1 ± 1.0 g, uDNX 18.4 ± 0.39 g, ANOVA *P* = 0.5467), as were heart (Sham 1.51 ± 0.15 g, UniNX 1.54 ± 0.17 g, uDNX 1.60 ± 0.16 g, *P* = 0.3775) and lung (Sham 2.61 ± 0.53 g, UniNX 2.55 ± 0.51 g, uDNX 2.49 ± 0.52 g, *P* = 0.8672).

### Blood metabolites and lipid metabolism

**Figure 3:**
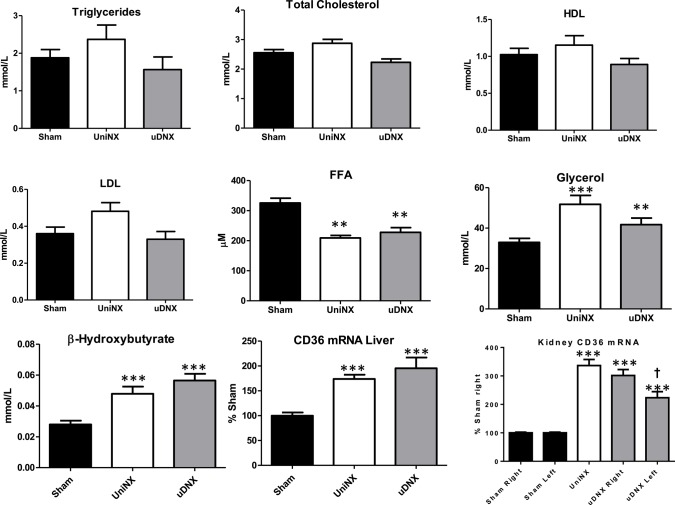
**Week 4 lipid metabolism**. Plasma triglycerides, total cholesterol, high density lipoprotein (HDL), low density lipoprotein (LDL), free fatty acids (FFA), glycerol, β-hydroxybutyrate, liver CD36 mRNA, and kidney CD36 mRNA in Sham operated, UniNX, and uDNX rats. Values represent means ± SE, *n* = 12/group for plasma and *n* = 8 in tissue. ^**^P < 0.01, ^***^P < 0.001 corresponds to UniNX/uDNX vs. Sham. ^†^*P* < 0.001 uDNXL (left) vs. Sham left.

**Figure 4:**
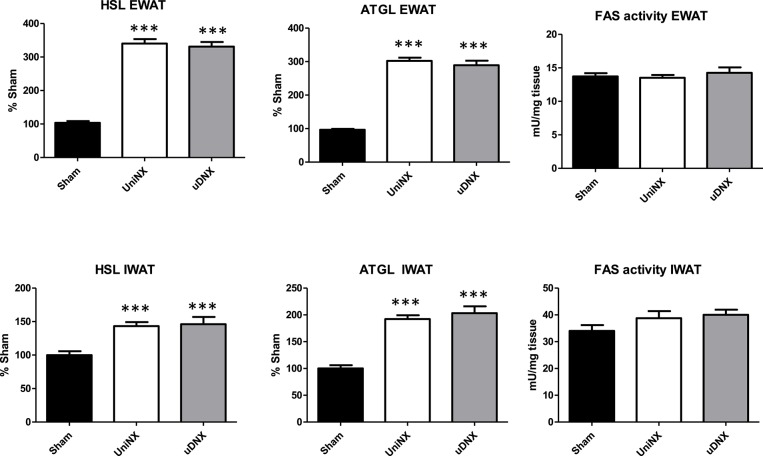
**Week 4 hormone sensitive lipase (HSL), adipose triglyceride lipase (ATGL) mRNA level and fatty acid synthase (FAS) activity in epididymal fat (EWAT) and Inguinal fat (IWAT) in Sham operated, UniNX and uDNX rats**. Values are means ± SE; *n* = 8/group. ^***^P < 0.001 corresponds to UniNX/uDNX vs. Sham.

No significant differences were seen in plasma glucose levels between the three groups (Sham 7.8 ± 0.2 mM, UniNX 7.9 ± 0.2 mM, and uDNX 7.9 ± 0.1 mM). There were no significance differences in triglycerides, total cholesterol, high density lipoproteins or low density lipoproteins (Figure 3). As previously reported, UniNX (Arsenijevic et al., 2015) decreased circulating free fatty acids (FFA) and increased both circulating β-hydroxybutyrate and glycerol. These changes also occurred in uDNX (Figure 3). CD36 mRNA was significantly increased in the liver in both UniNX and uDNX, and was also increased in the UniNX remnant kidney and in the intact uDNX controlateral kidney. Interestingly, CD36 in the uDNX denervated kidney was still elevated compared to Sham animals but not as high as in the uDNX contralateral kidney (Figure 3). CD36 mRNA was also elevated as percentage of Sham, in the heart (UniNX 151 ± 8%, *P* < 0.001; uDNX 144 ± 7%, *P* < 0.001) and in gastrocnemius muscle (UniNX 175 ± 8%, *P* < 0.001; uDNX 162 ± 11%, *P* < 0.001). Other tissues were not tested. Both UniNX and uDNX decreased EWAT and IWAT weight; this was associated with increased lipolysis (increased hormone sensitive lipase—HSL and adipose triglyceride lipase—ATGL mRNA levels) and was not related to changes in fatty acid synthase activity (Figure 4).

### Blood hormones

Blood hormones, aldosterone, angiotensin 2, corticosterone, ghrelin, glucagon like peptide-1, glucagon, insulin, leptin and T3 were not significantly different between Sham, UniNX and uDNX animals 4 weeks after surgery (Table 3).

**Table 3:** **Circulating plasma hormones 4 weeks post-surgery**.

Hormone	Sham	UniNX	uDNX
Aldosterone (ng/ml)	46.3 ± 1.5	46.8 ± 2.2	44.3 ± 1.7
Angiotensin-2 (ng/ml)	1.90 ± 0.10	1.80 ± 0.13	1.78 ± 0.08
Corticosterone (ng/ml)	23.5 ± 0.9	22.3 ± 1.3	22.1 ± 1.3
Ghrelin (pg/ml)	26.6 ± 1.5	29.3 ± 1.2	28.6 ± 2.2
GLP-1 (pg/ml)	5.28 ± 0.65	4.98 ± 0.90	4.64 ± 0.61
Glucagon (pg/ml)	286 ± 11	276 ± 12	279 ± 14
Insulin (ng/ml)	2.81 ± 0.26	2.63 ± 0.19	2.62 ± 0.20
Leptin (pg/ml)	469 ± 48	421 ± 51	417 ± 36
T3 total (ng/ml)	2.83 ± 0.26	2.65 ± 0.19	2.62 ± 0.20

*Values are average and SE: n = 12/group*.

### Serum and tissue cytokines

**Figure 5:**
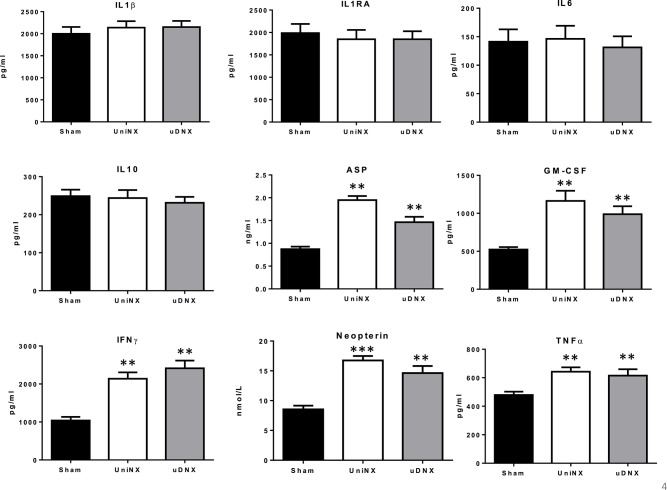
**Week 4 circulating inflammatory markers (IL1β, IL1RA, IL6, IL10, ASP, GM-CSF, IFNγ, Neopterin, and TNFα in Sham operated, UniNX and uDNX rats**. Values are means ± SE; *n* = 12/group, ^**^P < 0.01, ^***^P < 0.001 corresponds to UniNX/uDNX vs. Sham.

Circulating cytokine data were similar between UniNX and uDNX, including significant increases in ASP, GM-CSF, IFNγ, and TNFα, relative to Sham animals (Figure 5). As previously reported, UniNX increased macrophage activation (Arsenijevic et al., 2015) as evidenced by increased circulating neopterin and increased macrophage *in vitro* reactive oxygen production as estimated by NBT reduction. Similar responses were seen for uDNX animals. Peritoneal macrophages from UniNX and uDNX produced more reactive oxygen species (0.20 ± 0.01, *P* < 0.001 and 0.19 ± 0.01, *P* < 0.001 OD NTB, respectively) than Sham animals (0.12 ± 0.01 OD NTB).

**Figure 6:**
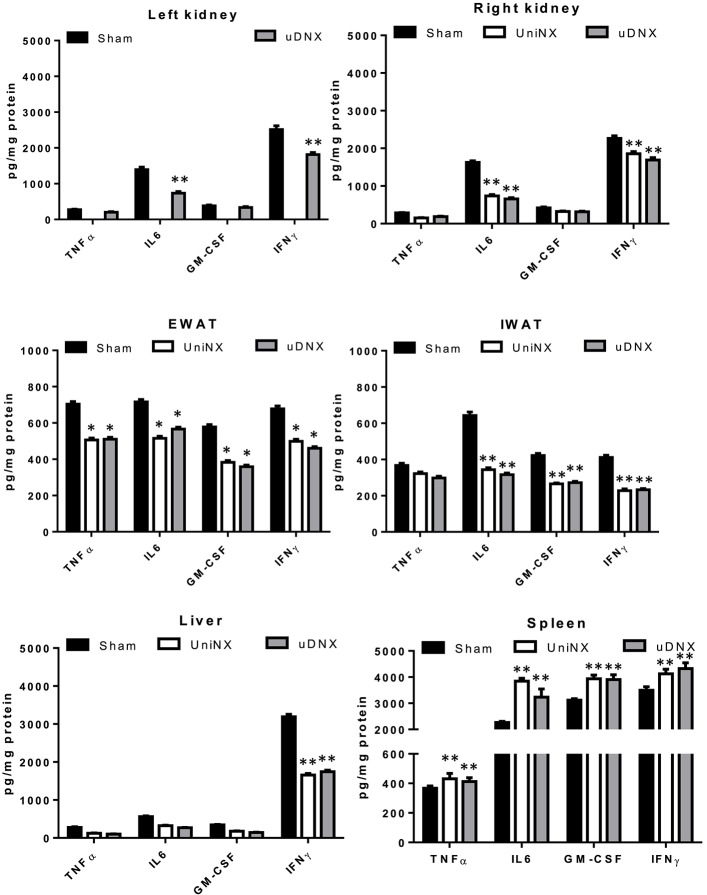
**TNFα, IL6, GM-CSF and IFNγ cytokine levels in kidney, epididymal fat pad (EWAT), inguinal fat pad (IWAT), liver and spleen on the 4^**th**^ week in Sham operated, UniNX and uDNX rats**. Values are means ± SE; *n* = 8/group. ^*^P < 0.05, ^*^^*^P < 0.01 corresponds to UniNX/uDNX vs. Sham.

Four selected cytokines (TNFα, IL6, GM-CSF, and IFNγ) were measured in various tissues at week 4. UniNX and uDNX decreased tissue cytokine protein levels in kidney, EWAT, IWAT, and liver compared to the Sham group (Figure 6). In contrast, these four cytokines were significantly increased in the spleen of UniNX and uDNX rats (Figure 6).

### Spleen weight, alpha2a adrenergic receptor, chemokines mRNA levels for monocyte chemoattractant protein-1 (MCP1)/regulated on activation, normal T cell expressed and secreted (RANTES)

**Figure 7:**
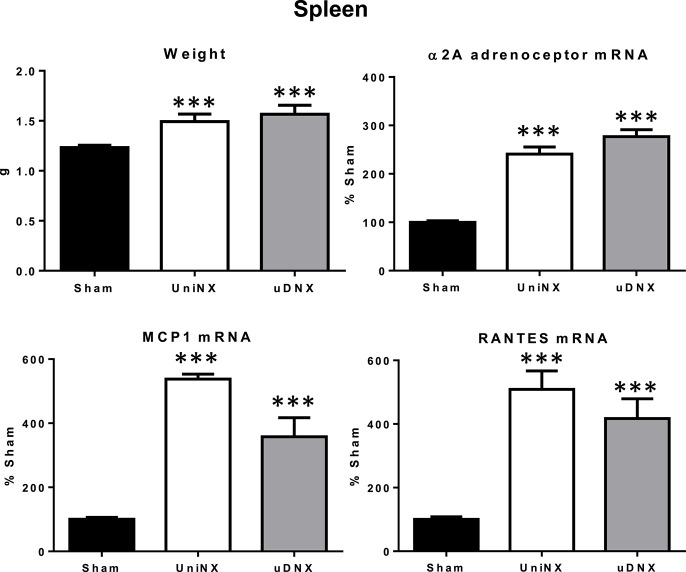
**Week 4 spleen values for weight, α_***2A***_ adrenoceptor mRNA levels, MCP1 mRNA levels and RANTES mRNA levels in Sham operated, UniNX, and uDNX rats**. Values are means ± SE; *n* = 8/group. ^***^P < 0.001 corresponds to UniNX/uDNX vs. Sham.

In both UniNX and uDNX we observed significant increases in spleen weight, pro-inflammatory alpha2A adrenergic receptor mRNA, monocyte chemokine MCP1 mRNA and lymphocyte chemokine RANTES mRNA (Figure 7).

### Noradrenaline content in selected tissues

**Figure 8:**
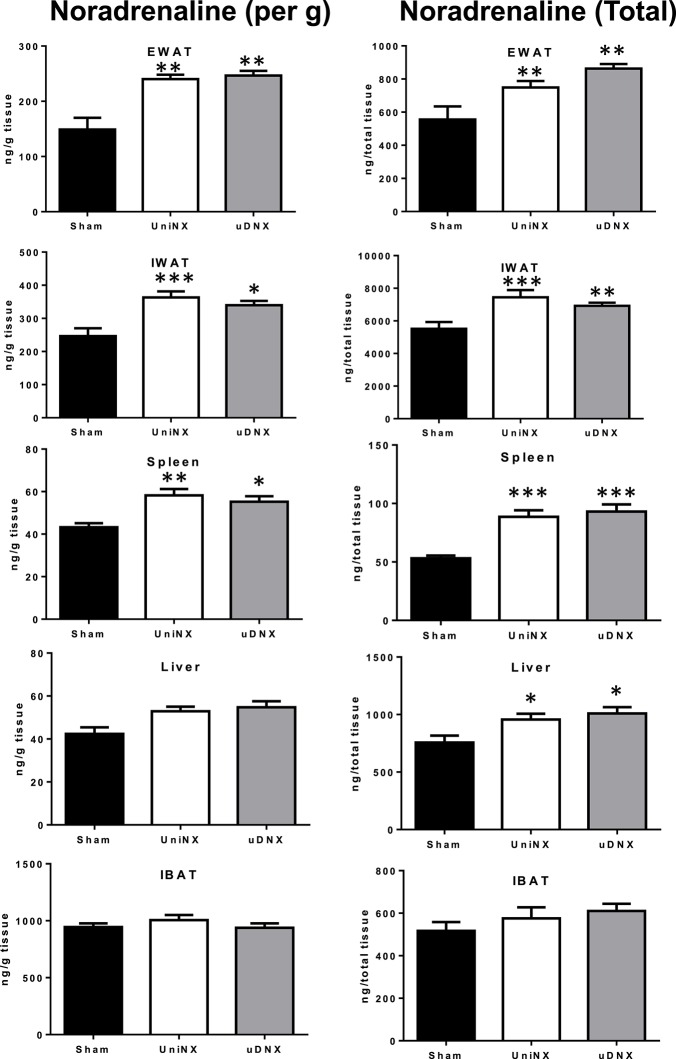
**Week 4 tissue noradrenaline levels expressed per g of tissue or total noradrenaline content in epididymal fat (EWAT), inguinal fat (IWAT), spleen, liver and interscapular brown adipose tissue (IBAT)**. Values are means ± SE; *n* = 8/group. ^*^P < 0.05, ^**^P < 0.01, ^***^P < 0.001 corresponds to Sham vs. UniNX or uDNX.

UniNX and uDNX increased noradrenaline content (expressed both by gram of tissue weight or as total organ amount) in epididymal fat, inguinal fat liver and spleen compared to Sham animals (Figure 8). There was no difference for liver noradrenaline content (expressed per g of tissue) between UniNX and uDNX animals, but it became significant when expressed as total organ amount. In interscapular brown adipose tissue (IBAT), there were no significant differences between the three groups in noradrenaline content (Figure 8) nor in protein levels of uncoupling protein 1 (Sham 1.39 ± 0.10 UCP1/beta-actin, UniNX 1.43 ± 0.07 UCP1/beta-actin, and uDNX 1.43 ± 0.08 UCP1/beta-actin, ANOVA *P* = 0.9366).

### Brainstem and hypothalamus melanocortin 4 receptor assessment by RT-PCR

**Figure 9:**
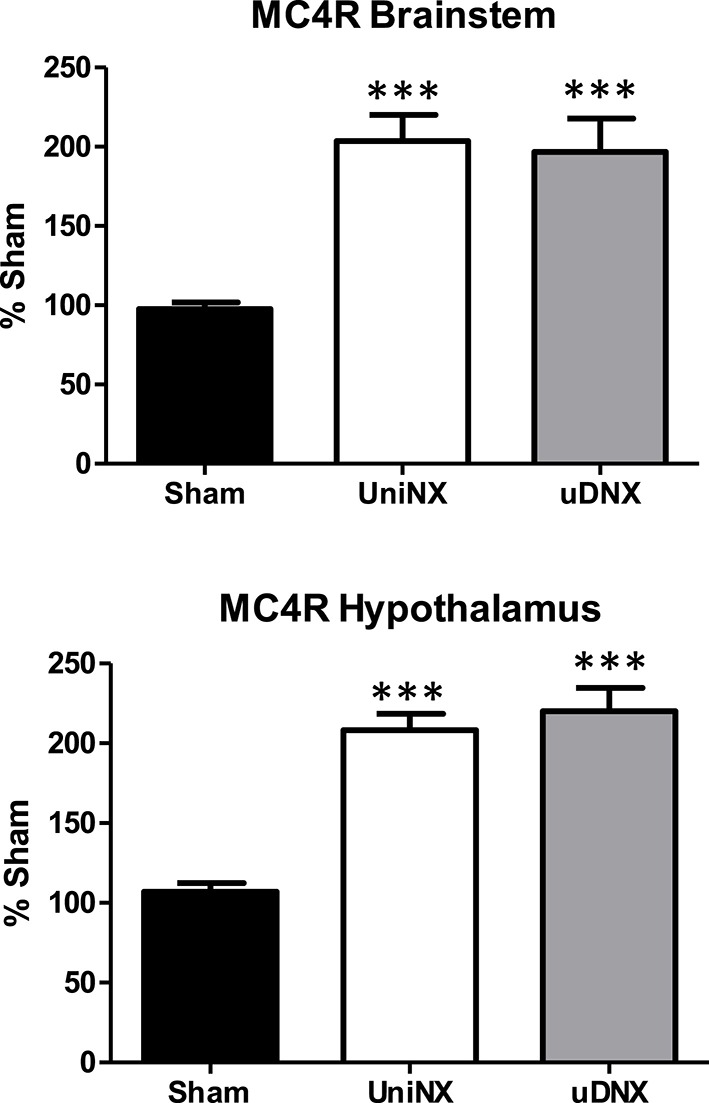
**Week 4 melanocortin 4 receptor (MC4R) mRNA levels in brainstem and hypothalamus in Sham operated, UniNX, and uDNX rats**. Values are means ± SE; *n* = 8/group. ^***^P < 0.001 corresponds to UniNX/uDNX vs. Sham.

Melanocortin 4 receptor (MC4R) mRNA was elevated in both the brainstem and the hypothalamus of UniNX and uDNX animals compared to Sham controls (Figure 9).

### Tissue FXR and SIRT1 protein levels

**Figure 10:**
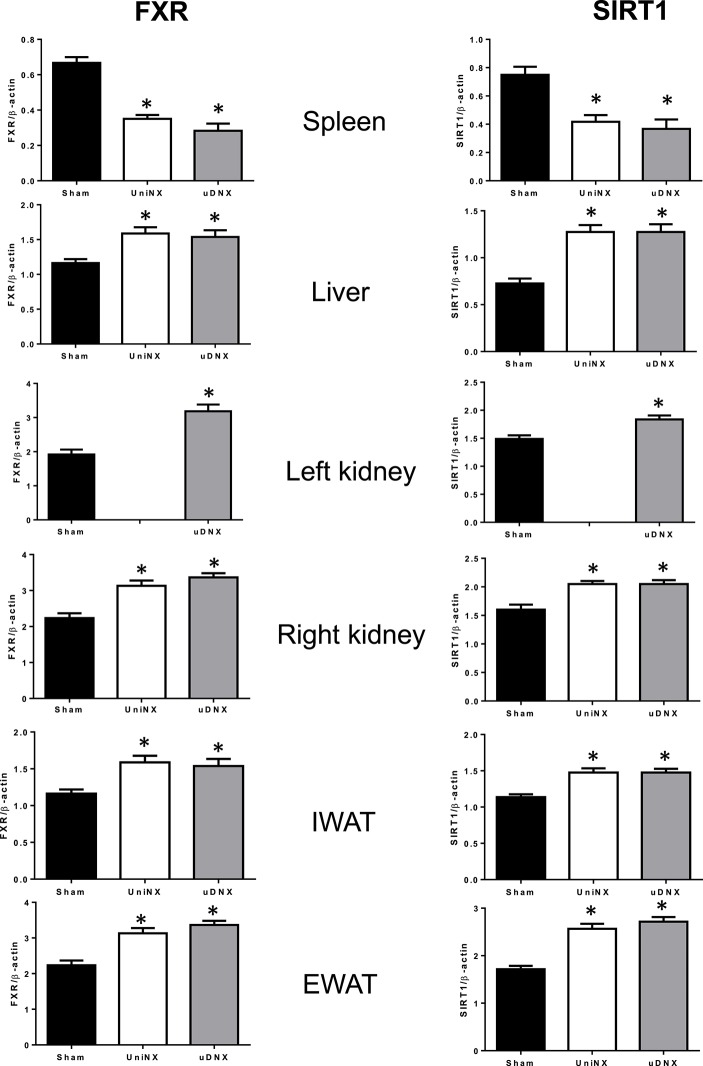
**Week 4 SIRT1 and FXR protein levels in spleen, liver, kidney, IWAT, and EWAT in Sham operated, UniNX, and uDNX rats**. Values are means ± SE; *n* = 8/group. ^*^P < 0.05 corresponds to UniNX/uDNX vs. Sham.

On week 4 SIRT1 and FXR proteins levels in IWAT, EWAT, kidney and liver were higher in UniNX and uDNX animals than in Sham controls. In sharp contrast, SIRT1 and FXR were lower in UniNX and uDNX spleen than in Sham (Figure 10).

## Discussion

We have previously described that UniNX, which results in a mild reduction in kidney function, leads to decreased total body fat and fat pad weights compared to Sham operated controls fed a fixed food intake diet. Decreases in fat pads were associated with increased lipolysis. This could not be explained by circulating hormones levels such as leptin, T3, insulin and ghrelin. However, a subset of circulating lipolytic cytokines TNFα, GM-CSF, and IFNγ were increased. These cytokines were elevated in the spleen of UniNX animals but were decreased in fat pads, kidney and liver. This suggested that these circulating cytokines might in part originate from the spleen. Interestingly, increased UniNX spleen cytokine levels were associated with decreased spleen anti-inflammatory regulators FXR/SIRT1 compared to Sham animals. In contrast, in other UniNX tissues such as fat pads, liver and kidney, FXR/SIRT1 were increased and cytokine levels were decreased compared to the Sham group (Arsenijevic et al., 2015).

The mechanism by which mild reduction in kidney function regulates lipolysis and low-grade inflammation is unknown. Since UniNX leads to removal of renal nerves on the side of surgery, we tested whether unilateral removal of renal nerve connections with the brain could contribute to the lipolytic and cytokine phenotype of UniNX. The major finding of the current study is that most of the phenotypes induced by UniNX were mimicked by unilateral renal denervation (uDNX).

Although uDNX did not reduce kidney function or increase kidney weight, uDNX like UniNX decreased both total body fat content and fat pad weight. This was most likely due to increased lipolysis in the two groups as suggested by the similar increases in lipases (HSL and ATGL mRNA) in EWAT and IWAT and by the increased circulating glycerol and β-hydroxybutyrate levels. The brain appears to be implicated in this process as both uDNX and UniNX increased the expression of the MC4 Receptor mRNA in both brainstem and hypothalamus. These brain areas have been previously implicated in MC4R—noradrenaline mediated lipolysis to fat pads (Song et al., 2005; Shrestha et al., 2010). Although tissue noradrenaline is not a measure of sympathetic nerve activity, we observed increased noradrenaline content in white fat pads in both UniNX and uDNX. Interestingly, no significant differences in noradrenaline or UCP1 content in IBAT were seen between the three groups. In addition, elevated noradrenaline content after uDNX/UniNX was observed in the liver and spleen. Increased noradrenaline in the liver, which has been shown to occur concomitantly with increased sympathetic activity in this tissue (Young and Landsberg, 1979) during cold exposure, could explain the increased FFA uptake by CD36 as is the case when rodents are subjected to cold exposure (Uchida et al., 2010). Noradrenaline and hepatic nerves have been shown to be involved in liver lipid metabolism (Wright et al., 2003; Carreno and Seelaender, 2004; Puschel, 2004; Jensen et al., 2013).

Noradrenaline in the spleen could modify the immune response (Elenkov et al., 2000), as it can modify cytokine production and release in an immune cell specific manner. TNFα originates from macrophage type cells, GM-CSF from endothelial cells and IFNγ from lymphocytes. Interestingly, increased spleen weight in uDNX/UniNX appears to be associated with increased chemokines MCP1 (for monocytes) and RANTES (for lymphocytes). Whether noradrenaline affects these immune cell subtypes in a specific manner for cytokine production and whether chemokines production is under control of adrenergic mechanisms remains to be determined for UniNX and uDNX. In addition, spleen cytokine regulation can be affected by adrenergic receptors. For example the up-regulation of the α_2A_-adrenergic receptor mRNA is implicated in spleen hyper-cytokine production in response to bacterial lipopolysaccharide (Miksa et al., 2009; Leong et al., 2010). Interestingly, we observed that uDNX/UniNX spleens, which have higher tissue noradrenaline content and cytokine levels, also have increased α_2A_-adrenergic receptor mRNA levels.

Furthermore, we have previously observed that FXR in non-splenic tissues are increased after UniNX (Arsenijevic et al., 2015) and this is now confirmed for uDNX. In line with the FXR anti-inflammatory action, the increased spleen cytokine levels (IL6, GM-CSF, IFNγ) were associated with decreased spleen FXR levels compared to Sham animals. Lack of FXR has been shown to enhance cytokine production in spleen (Mencarelli et al., 2009). Increased cytokines circulating could also play a role in increasing sympathetic outflow by acting on the brain (Shi et al., 2011; Capitanio and Cole, 2015).

Most of the work carried out on the function of renal nerves has employed bilateral renal denervation to demonstrate a role in blood pressure regulation. Bilateral renal denervation can also improve inflammation (Dorr et al., 2015), metabolic parameters and conditions associated with enhanced sympathetic activity (Bohm et al., 2013). Although there are many studies on the effects of bilateral denervation, there are far fewer studies on the effects of unilateral renal denervation (Bello-Reuss et al., 1975; Abdulla et al., 2008; Bischoff et al., 2015) and most of those have been acute studies conducted under anesthesia. Nevertheless, studies of chronic unilateral denervation in conscious animals have noted a tendency for BP to decrease in rats (Jacob et al., 2005), decreased inflammation in the denervated kidney during angiotensin-induced hypertension in mice (Xiao et al., 2015) and an improved heart function in a chronic rabbit heart failure model induced by rapid pacing in rabbits (Schiller et al., 2013). In unilateral renal injury model, unilateral renal denervation of the injured kidney improved the outcome, which was associated with a decrease in inflammation (Kim and Padanilam, 2015; Liang et al., 2015). In our study, chronic unilateral renal denervation led to a reduction in body fat associated with increased lipolysis in fat pads, increased brain MC4R mRNA levels and increased fat pad noradrenaline content.

Noradrenaline content was also elevated in the uDNX spleen as was the mRNA for the pro-inflammatory α_2A_-adrenergic receptor. A pro-inflammatory state of the spleen is also evidenced by the increase in spleen cytokines and the reduction of anti-inflammatory molecules such as FXR and SIRT1. Furthermore, the increase in spleen size was accompanied by elevations in chemokines for monocytes MCP1 and lymphocytes RANTES. Increased noradrenaline was also seen in the liver where metabolic factors were altered (i.e., increased CD36, FXR, SIRT1 levels). The increased noradrenaline in tissues appeared to be relatively specific since we did not observe increased noradrenaline/UCP1 in IBAT.

Our results suggest that uDNX induces changes in both immune and metabolic regulation. We speculate that uDNX leads to increased sympathetic nervous system activity, which may in part be mediated by hypothalamic pathways in which MC4R was increased. Noradrenaline tissue content may in turn be increased, leading to alterations in both tissue inflammation and metabolism. Since both metabolic and immune changes in uDNX closely resemble that seen in UniNX, we propose that most of the UniNX phenotype could be related to the unilateral removal of renal nerves following UniNX. These metabolic and immune changes may be relevant to kidney donors or subjects with mild renal dysfunction because mild dyslipidemia prior to kidney donation is a risk factor for predisposition to chronic kidney disease (Yoon et al., 2015). In addition, long-term consequences of live kidney donation may include a higher propensity of metabolic syndrome (Ferreira-Filho et al., 2007) and increased inflammatory markers (Yilmaz et al., 2015). However, we would like to point out that UniNX is a model of permanent nerve loss, whereas the uDNX is a transient model of nerve loss as reinnervation has been shown to occur within 3 months in the rat (Mulder et al., 2013).

In summary, experimental UniNX in rats promotes metabolic and immunological alterations by mechanisms that seem to implicate renal nerves and central pathways in which noradrenaline and FXR seem to play an important role. This suggests that peripheral and central neuronal components may be important in metabolic and immune changes induced by UniNX. The exact mechanisms involved remain to be determined. However, it is known that renal nerves are involved in complex reflex circuits with the brain. In addition to reno-renal reflexes (Kopp, 2015), it has been shown that there are reno-splenic reflexes (Moncrief et al., 2007) and adipose renal reflexes (Xiong et al., 2014). There is also overlap between different tissue neural circuits to the brain, in particular in both the hypothalamus and the brainstem, regions known to be involved in the regulation of metabolism and immunity (Pavlov and Tracey, 2012). Interestingly, we observed that MC4R gene expression is increased in the hypothalamus and brainstem to similar levels after both UniNX and uDNX. Consistent with our observation, in a more severe form of reduced kidney function in 5/6 nephrectomy, it has been shown that brain MC4R pathways are involved in decreasing body fat and regulating inflammatory cytokines (Cheung and Mak, 2012). We hypothesize that unilateral renal nerve removal results in changes of neural circuits which alters tissue metabolism and inflammatory state.

## Author contributions

Conceived and designed the experiments: DA, JM; Performed the experiments: DA, JC; Analyzed the data: DA, BF, AD, BV, JM; Wrote the paper: DA, JM; Edited manuscript: DA, AD, BV, JM.

### Conflict of interest statement

The authors declare that the research was conducted in the absence of any commercial or financial relationships that could be construed as a potential conflict of interest.

## Abbreviations

ASP: acetylation stimulating factor
ATGL: adipose triglyceride lipase
FXR: farnesoid x receptor
GM-CSF: granulocyte-macrophage colony stimulating factor
HSL: hormone sensitive lipase
IFNγ: interferon-gamma
UniNX: uninephrectomy
uDNX: unilateral renal denervation.
